# Genome, Proteome and Structure of a T7-Like Bacteriophage of the Kiwifruit Canker Phytopathogen *Pseudomonas syringae* pv. *actinidiae*

**DOI:** 10.3390/v7072776

**Published:** 2015-06-24

**Authors:** Rebekah A. Frampton, Elena Lopez Acedo, Vivienne L. Young, Danni Chen, Brian Tong, Corinda Taylor, Richard A. Easingwood, Andrew R. Pitman, Torsten Kleffmann, Mihnea Bostina, Peter C. Fineran

**Affiliations:** 1 Department of Microbiology and Immunology, University of Otago, PO Box 56, Dunedin 9054, New Zealand; E-Mails: rebekah.frampton@plantandfood.co.nz (R.A.F.); elpzacedo@gmail.com (E.L.A.); vivienne.young@otago.ac.nz (V.L.Y.); cheda856@student.otago.ac.nz (D.C.); tonyu031@student.otago.ac.nz (B.T.); corinda.taylor@otago.ac.nz (C.T.); 2 New Zealand Institute for Plant and Food Research Limited, Private Bag 4704, Christchurch 8140, New Zealand; E-Mail: andrew.pitman@plantandfood.co.nz; 3 Departamento de Genetica, Universidad de Extremadura, Badajoz 06080, Spain; 4 Otago Centre for Electron Microscopy, University of Otago, PO Box 56, Dunedin 9054, New Zealand; E-Mail: richard.easingwood@otago.ac.nz; 5 Centre for Protein Research, University of Otago, PO Box 56, Dunedin 9054, New Zealand; E-Mail: torsten.kleffman@otago.ac.nz

**Keywords:** biocontrol agent, kiwifruit, canker, *Pseudomonas syringae* pv. *actinidiae*, bacteriophage, cryo-electron microscopy, single particle reconstruction, genomics, proteomics, vB_PsyP_phiPsa17

## Abstract

*Pseudomonas syringae* pv. *actinidiae* is an economically significant pathogen responsible for severe bacterial canker of kiwifruit (*Actinidia* sp.). Bacteriophages infecting this phytopathogen have potential as biocontrol agents as part of an integrated approach to the management of bacterial canker, and for use as molecular tools to study this bacterium. A variety of bacteriophages were previously isolated that infect *P. syringae* pv. *actinidiae*, and their basic properties were characterized to provide a framework for formulation of these phages as biocontrol agents. Here, we have examined in more detail φPsa17, a phage with the capacity to infect a broad range of *P.* *syringae* pv. *actinidiae* strains and the only member of the *Podoviridae* in this collection. Particle morphology was visualized using cryo-electron microscopy, the genome was sequenced, and its structural proteins were analysed using shotgun proteomics. These studies demonstrated that φPsa17 has a 40,525 bp genome, is a member of the *T7likevirus* genus and is closely related to the pseudomonad phages φPSA2 and gh-1. Eleven structural proteins (one scaffolding) were detected by proteomics and φPsa17 has a capsid of approximately 60 nm in diameter. No genes indicative of a lysogenic lifecycle were identified, suggesting the phage is obligately lytic. These features indicate that φPsa17 may be suitable for formulation as a biocontrol agent of *P.* *syringae* pv. *actinidiae*.

## 1. Introduction

*Pseudomonas* *syringae* pv. *actinidiae* has recently emerged as a significant global pathogen [1], responsible for the bacterial canker of kiwifruit (*Actinidia* sp.). *P.* *syringae* pv. *actinidiae* was initially determined to be the bacterium responsible for this disease in Japan in 1989 [2]. The pathogen was subsequently detected in a number of kiwifruit growing countries, but its impacts could be effectively managed [3,4,5,6]. In 2008 however, a highly aggressive genotype of *P.* *syringae* pv. *actinidiae* emerged and has been detected in various countries in Europe, Asia, South America and Australasia [7,8,9,10,11,12,13,14]. These new *P.* *syringae* pv. *actinidiae* isolates are more aggressive and difficult to manage, with gold kiwifruit (e.g., *A. chinensis*) being particularly susceptible, although the green varieties (e.g., *A. deliciosa*) are also sensitive [15]. In November 2010, this aggressive strain of *P.* *syringae* pv. *actinidiae* was first detected in New Zealand [16] and now is estimated to be present in 87% of the kiwifruit orchards [17]. During infection, *P.* *syringae* pv. *actinidiae* can enter the plant through natural openings and lesions and causes leaf spotting, brown discoloration of buds, canker exudates on trunks and twigs, cane leader dieback and in severe cases, plant death [18,19].

Currently in New Zealand, *P.* *syringae* pv. *actinidiae* infections are being managed using a variety of approaches, which range from orchard practices and hygiene, through to new plant varieties (e.g., *A. chinensis* Gold3™) and streptomycin treatment [17]. Genetic analysis of the current worldwide pandemic strains demonstrates that they differ in only a few single nucleotide polymorphisms in their core genomes, with a variable content of genomic islands accounting for any major differences [20,21,22,23,24]. This relative homogeneity is suited for a phage therapy biocontrol approach, since the pathogen population is more easily targeted by specific phages [25,26]. Previously, we reported the isolation of a collection of ~250 phages that infect *P. syringae* pv. *actinidiae* [27]. Twenty four phages were analysed by host-range, cross-resistance profiling, negative staining and transmission electron microscopy, pulse-field gel electrophoresis and restriction digestion. The phages were all *Caudovirales* and representatives of the three major phage morphological groups were identified (*Myoviridae*, *Podoviridae* and *Siphoviridae*). Nine *Myoviridae* genomes were sequenced, one was assembled and an automated annotation performed. The eight other *Myoviridae* genome sequences were mapped to this complete genome and showed that they are similar, yet distinct. It is evident that parallel and complementary strategies are required for the long-term response to *P. syringae* pv. *actinidiae* and phages might be part of the approach. A greater understanding of the disease pathology of *P. syringae* pv. *actinidiae* is also desired and could be improved by new genetic tools. Phages have traditionally provided many such advances in genetic manipulation, including transducing vectors, integrative elements, restriction enzymes and other enzymes for making mutations [28,29]. Therefore, detailed analysis of *P. syringae* pv. *actinidiae* phages may reveal useful tools to analyse their host.

In this study, we have performed genomics, proteomics and morphological analyses of φPsa17. This phage was the only member of the *Podoviridae* that we isolated and was obtained from a wastewater sample in Tahuna, Dunedin, NZ, an area with no known or detected *P. syringae* pv. *actinidiae* [27]. The lack of *P. syringae* pv. *actinidiae* suggests that the native host of φPsa17 is another related bacterium. Indeed, φPsa17 was shown to have a broader host range than many of the other phages isolated in our original study, infecting Italian, Japanese and South Korean strains, and some isolates from pre-2008 outbreaks in addition to other New Zealand pseudomonads [27]. Here, we show that φPsa17 is a member of the *Caudovirales* order, *Podoviridae* family, *Autographivirinae* subfamily and *T7likevirus* genus in terms of both genomic content, arrangement and capsid structure and protein composition.

## 2. Materials and Methods

### 2.1. Materials, Bacterial Strains and Culture Conditions

Phages were grown on *Pseudomonas* *syringae* pv. *actinidiae* ICMP 18800, which was isolated from *A. deliciosa* kiwifruit plants in Paengaroa, New Zealand in 2010 [20]. Bacteria were grown in Nutrient Broth (NB; 5 g L^−1^ peptone, 3 g L^−1^ yeast extract, and 5 g L^−1^ NaCl) at 25 °C or on solid NB medium containing 1.5% (w v^−1^) agar. Unless stated otherwise, soft medium (top) agar (0.5% w v^−1^) was used for bacteriophage titrations. Phage buffer was composed of 10 mM Tris-HCl pH 7.4, 10 mM MgSO_4_, and 0.01% (w v^−1^) gelatin. The CHCl_3_ used in this study was saturated with sodium hydrogen carbonate.

### 2.2. Phage Lysate Preparation

Phage φPsa17 lysates were prepared by serial dilution in phage buffer and 100 μL was added to 4 mL soft NB agar containing 100 μL of a *P. syringae* pv. *actinidiae* overnight culture. The soft agar containing the phage and bacteria was poured onto plates and incubated overnight at 25 °C. Lysates were prepared from plates with almost confluent lysis by removing the soft agar overlay using a sterile glass slide and combining with 3 mL of phage buffer that was used to wash the plate. The agar was mixed with 500 μL CHCl_3_, vortexed for 2 min, incubated at room temperature for 30 min, centrifuged in fluorinated ethylene propylene (FEP) JA20 tubes at 2442 *g* for 30 min at 4 °C and the supernatant was retained. The phage lysates were stored at 4 °C with 100 μL CHCl_3_ added. Phage lysates were titred using the same double agar overlay method as described above. These lysates were used for DNA preparations, sequencing and inoculation of larger cultures for higher-purity and higher-titre preparations.

### 2.3. Phage Genome Sequencing, Assembly and Annotation

DNA from φPsa17 was isolated as described previously and the concentration was determined using a nanodrop ND1000 [27]. The DNA was further purified and eluted using the column binding, washing and elution steps of the Qiagen DNeasy Blood & Tissue Kit (Venlo, The Netherlands). Genome sequencing was performed by New Zealand Genomics Ltd and the libraries were prepared using the Illumina TruSeq DNA Sample Preparation v2 kit (San Diego, CA, USA). Libraries were quantified using a Bioanalyzer 2100 DNA 1000 chip (Agilent; Santa Clara, CA, USA) and the Qubit Fluorometer using the dsDNABR kit (Life Technologies; Carlsbad, CA, USA). MiSeq 150 bp paired-end sequencing was performed and de-multiplexed using the ea-utils suite of tools [30]. The φPsa17 phage was assembled into one contig using 1,101,120 reads and Geneious v6.1.6 [31] (*de novo* assembly using default settings with increased sensitivity). The contig ends overlapped allowing a single circular phage scaffold assembly to be generated. The reads were mapped back to the assembly for validation and approximately 4,100-fold coverage was achieved. The genome was linearised after identification of direct terminal repeats due to read ends and increased coverage across the repeat region (see Results and Discussion). Automated annotation of φPsa17 was performed using RAST [32], and the presence of tRNAs was assessed using tRNAscan-SE [33] and ARAGORN [34]. CDSs were further analysed using BLAST [35] with a particular focus on members of the related *T7likevirus* genus for manual curation. Putative phage promoters and RBSs were identified and then manually curated by extracting the 100 bp upstream of the predicted CDSs [36] and looking for motifs using MEME [37]. Rho-independent terminators were predicted using ARNold (Erpin and RNAmotif) [38,39,40]. The presence of putative *P. syringae* pv. *actinidiae* promoter sequences was investigated using BPROM (linear discriminant function (LDF) threshold ≥3) [41] and Neural Network Promoter Prediction (minimum promoter score 0.9) [42]. Pairwise phage comparisons were performed using blastn within Easyfig [43]. The φPsa17 genome has been deposited in GenBank under accession number KR091952.

### 2.4. Purification of φPsa17 for Proteomics

*P. syringae* pv. *actinidiae* was grown overnight in 20 mL NB and used to inoculate 500 mL of NB in a 2 L baffled flask. Bacteria were grown with shaking at 160 rpm at 25 °C until an OD_600_ ≈ 0.3 (~5 × 10^8^ cfu mL^−1^) and φPsa17 added at a multiplicity of infection (MOI) of 0.0001. Infection was continued for 5 h (160 rpm at 25 °C) and the culture stored at 4 °C overnight. For PEG precipitation, cultures containing φPsa17 were brought up to room temperature and 20 g of NaCl was added per 500 mL culture and dissolved. Cultures were stored on ice for 1 h and cell debris was removed by centrifugation at 11,000 *g* for 10 min at 4 °C. The supernatant was decanted, PEG6000 was added (10% w v^−1^ final concentration), dissolved by stirring at room temperature and stored on ice overnight to precipitate the phage. The precipitate was pelleted by centrifugation at 11,000 *g* for 20 min at 4 °C, the supernatant discarded and the tubes dried by inversion for 5 min. The phage pellet was resuspended gently in 600 μL of phage buffer by soaking at room temperature for 1 h. The PEG and cell debris were extracted from the phage suspension by adding an equal volume of CHCl_3_, vortexing for 1 min and centrifuging at 4000 *g* for 15 min at 4 °C. The aqueous phase containing phages, was recovered. Phages were titred and the protein concentration determined using the BCA Protein Assay Kit (Thermo Fisher Scientific; Waltham, MA, USA).

Phages were further purified using a stepwise CsCl gradient based on a method previously described [44]. The gradients were prepared in Beckman SW32.1 tubes by subsequently underlaying 1.5 mL of each 1.33, 1.45, 1.6 and 1.7 g/cm^3^ CsCl solution. Phages were gently added (11 mL, containing 0.5 g/mL CsCl to avoid osmotic shock) on top of the 1.33 g/cm^3^ CsCl. The tubes were centrifuged at 140,000 *g* for 3 h at 4 °C. The opalescent phage band was collected using a glass pasteur pipette and dialysed (1000 kDa MWCO), twice for 30 min and once overnight, against 250 volumes (500 mL) of distilled water to remove CsCl. Phages were concentrated by vacuum centrifugation for 2 h at medium temperature and the titre was determined.

### 2.5. Proteomics of φPsa17

*Sample preparation*: For proteomics of the structural phage proteins, purified phages were combined with 4 × SDS loading dye (40 mM Tris-HCl (pH 6.8), 40% glycerol, 4 mM EDTA, 2.5% SDS, 0.2 mg mL^−1^ bromophenol blue), boiled for 5 min and separated by 12% SDS-PAGE (polyacrylamide gel electrophoresis). The gel lane of separated φPsa17 proteins was fractionated into eight molecular weight fractions and subjected to in-gel digestion with trypsin [45] using a robotic workstation for automated protein digestion (DigestPro Msi, Intavis AG; Cologne, Germany). Extracted tryptic peptides were concentrated using a centrifugal vacuum concentrator.

*Protein identification by liquid chromatography-coupled tandem mass spectrometry (LC-MS/MS):* The tryptic peptides of each fraction were reconstituted in 15 µL of 5% (v v^−1^) acetonitrile, 0.2% (v v^−1^) formic acid in water and 5 µL were injected per run into an Ultimate 3000 nano-flow uHPLC-System (Dionex Co, Thermo Fisher Scientific; Waltham, MA, USA) that was in-line coupled to the nanospray source of a LTQ-Orbitrap XL mass spectrometer (Thermo Scientific; Waltham, USA). Peptides were separated on an in-house packed emitter-tip column (75 µm ID PicoTip fused silica tubing (New Objectives, Woburn, MA, USA) filled with C-18 material (3 µm bead size) to a length of 12 cm). Each fraction was analysed twice using slightly different LC-MS/MS conditions to improve protein identification and sequence coverage. For the first analysis, acetonitrile (ACN) gradients were formed from 5% solvent B (0.2% (v v^−1^) formic acid in ACN) in solvent A (0.2% (v v^−1^) formic acid in water) over 30 min, followed by increasing solvent B first to 45% over 6 min and then to 90% over 5 min. All steps were performed at a flow rate of 500 nL min^−1^ and followed by column washing and re-equilibration. For the second analysis, the durations of the first (from 5% to 25% solvent B) and second (from 25% to 40% solvent B) step were changed to 60 min and 10 min, respectively.

The orbitrap mass analyser was operated in a mass range between m/z 400 and 2000 at a resolution of 60,000 at *m/z* 400 for the acquisition of precursor ion spectra. Preview mode for FTMS master scan was enabled to generate the precursor mass lists. The strongest six (analysis 1) or eight (analysis 2) precursor ion signals were selected for CID (collision-induced dissociation)-MS/MS in the LTQ ion trap at a normalised collision energy of 35%. Dynamic exclusion was enabled with two repeat counts during 90 s followed by an exclusion period of 120 s.

*Data analysis*: Raw spectra were processed through the Proteome Discoverer software (Thermo Fisher Scientific, Waltham, MA, USA) using default settings to generate peak lists. Peak lists were then searched against a combined amino acid sequence database containing all φPsa17 sequence entries (GenBank accession KR091952, 49 entries) integrated into the full SwissProt/UniProt sequence database (downloaded July 2014; 546,000 entries) using the Sequest HT (Thermo Fisher Scientific; Waltham, MA, USA), Mascot (www.matrixscience.com) and MS Amanda [46] search engines. The same search settings were used for all three search engines allowing for semi-tryptic cleavages with a maximum of three missed cleavage sites and carboxyamidomethyl cysteine, oxidised methionine and deamidated asparagine and glutamine as variable modifications. The mass tolerance threshold was 10 ppm and 0.8 Da for precursor ions and fragment ions respectively.

The Percolator algorithm [47] was used to estimate the False Discovery Rate (FDR). Peptide hits were filtered for a strict FDR of *q* < 0.01. Additional peptide score filters for each search engine were applied to eliminate very low scoring peptide hits that may have passed the Percolator FDR filter. The following score thresholds for positive peptide identification were applied: Mascot peptide score of >20, MS Amanda score >100 and the default charge state (z)-dependent Sequest HT cross correlation scores (XCorr) that is 2.0 (z = 2), 2.25 (z = 3), 2.5 (z = 4), 2.75 (z = 5) and 3.2 for all other charge states. We only accepted protein identifications with two or more significant peptide hits that were consistently assigned to the same protein by all three search engines.

### 2.6. Purification of φPsa17 for Cryo-Electron Microscopy

Phage lysates were prepared as described in *Section 2.2*, with the exception that 2.5 mL of NB with 0.75% w v^−1^ agar was used in the top overlay and the harvested plates were rinsed with 1 mL of phage buffer. Multiple samples were prepared and pooled to generate 2 × 11 mL of high-titre phage stock that was then further purified using CsCl gradients as described in Section 2.4. The phages were dialysed (3.5 kDa MWCO) once for 8 h and once overnight in 800 mL water to remove CsCl.

### 2.7. Transmission Electron Microscopy and Cryo-Electron Microscopy

High-titre phage lysates were prepared for transmission electron microscopy (TEM) on 300-mesh copper grids carbon coated and stained with 2% phosphotungstic acid solution (pH 7) for 1 min, as described previously [27]. Residual liquid was blotted away with filter paper. Transmission electron micrographs were recorded using a Philips CM100 TEM (Philips/FEI Corporation, Eindhoven, The Netherlands) operated at 100 kV at a magnification of 66,000.

Cryogenic specimens were prepared by first applying 3 µL of purified virus on glow discharged Quantifoil holey carbon grids (Quantifoil Micro Tools GmbH, Grossloebichau/Jena, Germany). The excess buffer was blotted and the grid was flash plunged into liquid ethane using a Leica KF80 cryo fixation device (C. Reichert Optische Werke AG, Vienna, Austria). Frozen-hydrated specimens were stored in liquid nitrogen until loaded onto a Gatan 914 Cryoholder (Pleasanton, CA, USA). A JEOL JEM2200FS microscope (JEOL Ltd, Tokyo, Japan) operated at 200 kV was used for visualisation using minimal dose conditions with an electron dose less than 20 electrons/Å^2^. An in-column omega energy filter was used to improve image contrast by zero-loss filtering with a slit width of 25 eV. Automated data collection was carried out using *SerialEM* software [48]. The micrographs were recorded at a defocus between 1 and 3 µm, on a 4 × 4 k CMOS camera (TVIPS; Gauting, Germany) at a calibrated magnification of 50,000 corresponding to a pixel size of 3.12 Å.

*Image processing.* Individual phage particles were selected from micrographs using the *E2BOXER* programme [49]. Contrast Transfer Function parameters were calculated using *CTFFIND3* [50], and micrographs with poor CTF estimates were discarded. Orientation, classification and refinement were done using *Relion* [51]. The resultant map was visualised using *Chimera* [52].

## 3. Results and Discussion

### 3.1. Podovirus φPsa17 Genome Sequence

**Figure 1:**
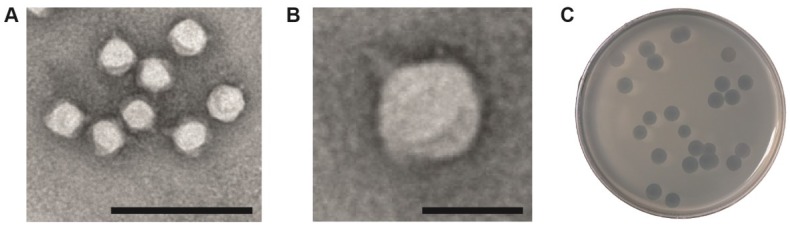
(**a**) and (**b**) Transmission electron micrographs of φPsa17 by negative stained TEM; (**c**) Plaque morphology of φPsa17 on *P. syringae* pv. *actinidiae* ICMP18800 in nutrient broth with 0.5% agar; In (**a**) and (**b**) the scale bars represent 200 nm and 50 nm, respectively.

φPsa17 was identified as a member of the *Caudovirales* order and *Podoviridae* family when observed by negative staining and transmission electron microscopy (Figure 1A,B). Plaques formed by φPsa17 are large and clear with a turbid ring on 0.5% agar, with a total diameter of ~4–5 mm (Figure 1C). To characterise φPsa17, which was the only podovirus we previously isolated that infected *P. syringae* pv. *actinidiae*, genome sequencing was performed. The genome was sequenced using Illumina MiSeq (150 bp paired-end) and assembled into a linear genome with overlapping ends. By mapping the reads back to a circularised assembly, we observed an increased read depth in one region, indicating possible terminal redundancy. Interrogation of the read mapping revealed defined ends with increased sequence depth, which enabled the prediction that the genome was linear dsDNA with two 242 bp terminal repeats.

**Figure 2:**
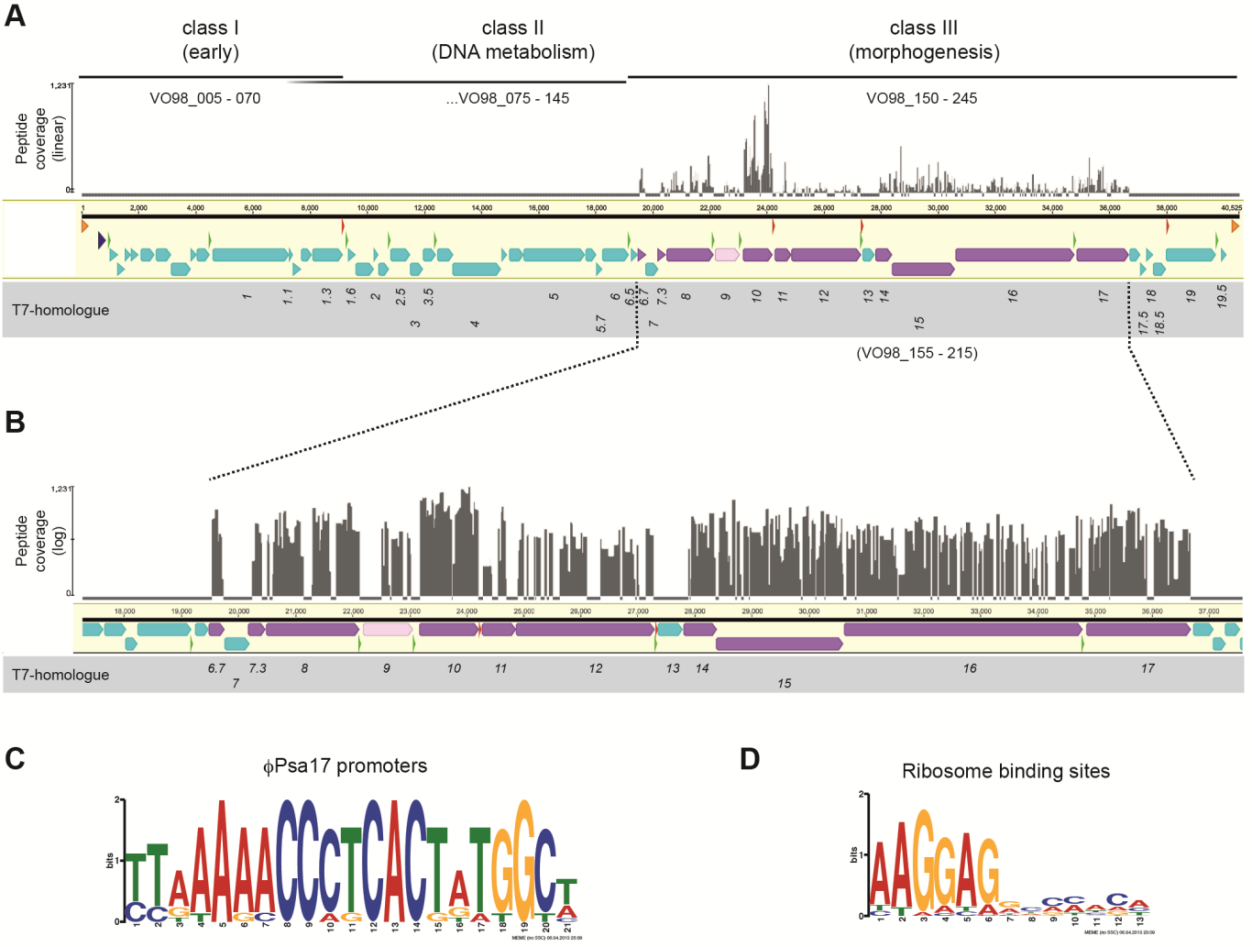
(**a**) The φPsa17 genome sequence and organisation is similar to members of the *T7likevirus* genus. The linear genome is 40,525 bp with terminal repeats (orange arrows). Predicted CDS are shown as arrows in cyan (or purple/pink if identified by proteomics). A putative *Pseudomonas* promoter early in the genome is indicated (dark blue triangle) and 11 predicted phage RNAP-dependent promoters are depicted (green triangles). Four putative Rho-independent transcriptional terminators are shown (red triangles). Putative classes of genes (I, II & III), based on both homologues in T7 and genome organisation [53], are indicated and locus tags provided. “Coverage” indicates peptide mapping identified by proteomics (linear scale); (**b**) Expanded view of the structural proteome mapping data with “coverage” on a log scale; Images in (**a**) and (**b**) were prepared using Geneious v8.1.2 [31] and Adobe Illustrator; Sequence logos of the (**c**) 11 phage promoters and (**d**) 47 ribosome binding sites that were predicted. Logos were generated using MEME [37].

The genome is 40,525 bp, linear, terminally redundant and has a 57% G + C content (Figure 2A,B). The genome was annotated, indicating the presence of 49 putative coding sequences (CDSs) (Table 1). φPsa17 contains no predicted tRNAs, which might be explained by its G + C content being similar to the host bacterium (57% *vs.* 58% for *P. syringae* pv. *actinidiae*). Interestingly, we recently sequenced φPsa374, a *Myoviridae* that infected *P. syringae* pv. *actinidiae*, which had a G + C content of 47.4% and contained 11 tRNAs [27].

**Table 1:** Putative genes of φPsa17 and their homologues in T7 and conserved domains.

Locus Tag	T7 Homologue	Description	Start	End	Length (bp)	Domains or PHA#
VO98_005		hypothetical	993	1262	270	
VO98_010		hypothetical	1259	1513	255	
VO98_015		hypothetical	1513	1710	198	
VO98_020		hypothetical	1715	1990	276	
VO98_025		hypothetical	2078	2557	480	
VO98_030		hypothetical	2588	3124	537	
VO98_035		hypothetical	3121	3825	705	Pfam13640
VO98_040		hypothetical	3825	4028	204	
VO98_045		hypothetical	4030	4470	441	
VO98_050	gp1	DNA-directed RNA polymerase (EC 2.7.7.6)	4598	7255	2658	PHA00452
VO98_055	gp1.1	hypothetical	7269	7406	138	
VO98_060		hypothetical	7403	7675	273	
VO98_065		hypothetical	7675	8061	387	
VO98_070	gp1.3	ATP-dependent DNA ligase	8073	9137	1065	PHA00454
VO98_075	gp1.6	hypothetical	9331	9588	258	PHA00455
VO98_080		hypothetical	9585	10,232	648	ABC_ATPase superfamily
VO98_085	gp2	host RNA polymerase inhibitor	10,229	10,396	168	PHA00457
VO98_090		hypothetical	10,393	10,758	366	
VO98_095	gp2.5	T7-like ssDNA-binding	10,812	11,513	702	PHA00458
VO98_100	gp3	T7-like endonuclease (EC 3.1.21.2)	11,513	11,956	444	Phage_endo_I superfamily
VO98_105	gp3.5	lysozyme, N-acetylmuramoyl-L-alanine amidase (EC 3.5.1.28)	11,959	12,399	441	PGRP superfamily
VO98_110		hypothetical	12,469	13,008	540	PolyA_pol superfamily
VO98_115	gp4	T7-like DNA primase/helicase	12,995	14,686	1692	
VO98_120		hypothetical	14,705	14,902	198	
VO98_125		hypothetical	14,971	15,480	510	
VO98_130	gp5	T7-like DNA Polymerase (EC 2.7.7.7)	15,491	17,638	2148	DNA_pol_A superfamily
VO98_135		hypothetical	17,651	18,031	381	
VO98_140	gp5.7	hypothetical	18,024	18,233	210	PHA00422
VO98_145	gp6	exonuclease	18,230	19,174	945	PHA00439
VO98_150	gp6.5	hypothetical	19,243	19,485	243	DUF2717
VO98_155	gp6.7	T7 virion protein	19,488	19,760	273	PHA00441
VO98_160	gp7	hypothetical	19,757	20,200	444	PHA01807
VO98_165	gp7.3	tail assembly	20,172	20,474	303	PHA00437
VO98_170	gp8	collar/T7-like head-to-tail connector	20,489	22,120	1632	PHA00670
VO98_175	gp9	capsid and scaffold	22,189	23,064	876	PHA00435
VO98_180	gp10	major capsid protein	23,164	24,207	1044	PHA00201 (PHA02004 superfamily)
VO98_185	gp11	T7-like tail tubular protein A	24,271	24,858	588	PHA00428
VO98_190	gp12	T7-like tail tubular protein B	24,868	27,294	2427	
VO98_195	gp13	protein inside capsid A	27,353	27,787	435	PHA00432
VO98_200	gp14	protein inside capsid B	27,798	28,385	588	PHA00101
VO98_205	gp15	protein inside capsid C	28,378	30,594	2217	PHA00431
VO98_210	gp16	protein inside capsid D	30,607	34,785	4179	PHA00638
VO98_215	gp17	tail fibre	34,848	36,677	1830	PHA00430
VO98_220		hypothetical	36,717	37,073	357	
VO98_225	gp17.5	holin, class II	37,073	37,288	216	PHA00426
VO98_230	gp18	T7-like DNA packaging protein A, small terminase subunit	37,285	37,542	258	PHA00425
VO98_235	gp18.5	endopeptidase (EC 3.4.-.-), lambda Rz-like	37,542	37,991	450	PHA00276
VO98_240	gp19	DNA packaging, large terminase subunit	37,991	39,739	1749	Pfam03237
VO98_245	gp19.5	hypothetical	39,919	40,092	174	

### 3.2. Transcriptional Organisation of the φPsa17 Genome

The φPsa17 genome has genetic and organisational similarity to other members of the *Autographivirinae* subfamily and *T7likevirus* genus of phages (see Section 3.3). Based on the similarity to T7, we have proposed an organisation of the φPsa17 genome into three classes (I, II and III), representing the early, DNA metabolism and morphogenesis genes, respectively (Figure 2A) [53]. DNA injection is likely to occur in a similar manner to T7 and involve a first stage where ~850 bp is injected. One promoter that is potentially recognised by the *P. syringae* pv. *actinidiae* RNAP was identified in this early genomic region (positions 610–637; TTGACA-N_16_-TAAGA (Figure 2A; dark green triangle). This might be a strong promoter since a 16 nt spacing in *Pseudomonas aeruginosa* promoters enhances transcription [54]. Visual inspection of the φPsa17 genome identified a TTGACA-N_18_-TATGCG sequence (position 275–304) that might also be involved in initiating host-dependent transcription of the early genes. These host promoters are likely to act similarly to the three major *Escherichia coli*-dependent promoters (A1-A3) in T7 to assist in the expression, and transcription-dependent translocation, of a further ~7 kb of the phage genome into the host bacterium, which consists of the class I early genes [53].

Eleven putative phage promoters were identified upstream of CDSs in φPsa17 (Figure 2A,C; green triangles), and are predicted to drive the expression of the class II and III genes. A sequence logo of these phage RNA polymerase (RNAP)-dependent promoters was generated, revealing the 21 bp consensus 5′-TTAAAAACCCTCACTATGGCT-3′ (Figure 2C). This promoter is similar to the consensus identified by Kovalyova and Kropinski for a related *Pseudomonas putida* phage gh-1 (5′-TAAAAACCCTCACTRTGGCHSCM-3′) [55].

An early CDS, locus tag VO98_050, encodes a DNA-dependent RNA polymerase (EC 2.7.7.6), which is the equivalent of gene *1* in *E. coli* phage T7 and is likely to be responsible for transcription from these phage promoters. Based on the similarity to T7, it is probable that the φPsa17 RNAP drives the translocation of the remainder of the genome into *P. syringae* pv. *actinidiae*. One reason for this multi-stage slow genome injection in T7 is to enable the production of defense proteins, in particular gp0.3 (Ocr), which mimics DNA and inhibits Type I restriction endonucleases [56]. We did not detect a homologue of gp0.3 in φPsa17, but it is possible that other poorly characterised class I and II genes might be performing similar functions (Table 1). Using structural searches (Phyre2 [57]) and HMM approaches (Phmmer [58]) on VO98_005 to VO98_045, we were unable to predict any additional domains for these predicted proteins. The shift from class II to III gene expression requires the action of gene *3.5* in T7 (aka lysozyme) [53] and φPsa17 possesses a homologue (VO98_105). Finally, four putative transcriptional terminators were detected and, like T7, “good” ribosome binding sites were detected upstream of almost all (*i.e.*, 47) of the CDSs and had the consensus 5′-AAGGAG-3′ (Figure 2C). Therefore, the genome organisation of φPsa17 is highly similar to T7 and other close *T7likevirus* relatives (see Section 3.3).

### 3.3. φPsa17 is a T7likevirus Similar to φPSA2 and gh-1

**Figure 3:**
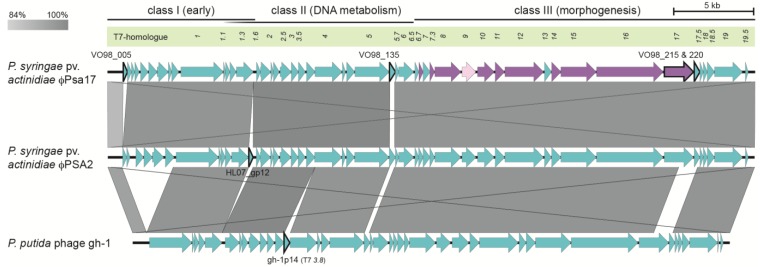
Genome comparison of *P. syringae* pv. *actinidiae* φPsa17 with φPSA2 and *P. putida* gh-1 phages. Pairwise phage comparisons were performed using blastn within Easyfig and the grayscale indicates genes with 84%–100% nt identity [43]. Classes of genes and their T7-homologues are provided (as shown in Figure 2). Genes present in one genome, but lacking or having lower than 84% identity in others, lack gray regions indicating identity. Genes discussed in the text are outlined in black and the locus tags provided. The conservation of terminal repeats is visible as the large crossed gray lines. Accession numbers of input genomes were; φPsa17 (KR091952), φPSA2 (NC_024362) [59] and *P. putida* gh-1 (NC_004665) [55].

Both the organisation and sequence similarity of the genome to existing phages strongly support the assignment of φPsa17 as a member of the *T7likevirus* genus. φPsa17 is highly similar to the well-annotated *P. putida* phage gh-1 (93% nt identity) (Figure 3) [55]. However, φPsa17 has an additional ~3.5 kb in the early region, after the terminal repeat and prior to the T7-like RNA polymerase (gene *1*). Most members of the *T7likevirus* genus have genes in this region and the absence of genes in gh-1 is the exception [55]. Surprisingly, φPsa17, which was the first podovirus isolated that infects *P. syringae* pv. *actinidiae* [27], is nearly identical in terms of organisation and sequence (96% nt identity) to a recently sequenced *P. syringae* pv. *actinidiae* phage, φPSA2 (Figure 3) [59]. Both phages were isolated from sewerage, φPsa17 in Dunedin, New Zealand [27] and φPSA2 from Rome, Italy [59]. There are a number of differences between the two phages. Firstly, each phage has a single CDS that is absent in the other genome. The first CDS in φPsa17 (VO98_005) is absent in both φPSA2 and phage gh-1. This is a hypothetical protein with no known function, but homologues are present in other phages that infect members of the Enterobacteriaceae. The most closely related protein is encoded from the chromosome of *Sinorhizobium meliloti* AK83. Conversely, φPSA2 contains a gene (locus tag HL07_gp12; *orf12* [59]) that is absent from φPsa17 (and gh-1). The function of this protein is unknown and no similar proteins were detected using tBLASTn. Another CDS of interest from the comparative analysis (Figure 3) is φPsa17 VO98_135, which appears different from those present in φPSA2 and gh-1. However, this is an artifact of the cut-off in Figure 3 and these genes are homologues of unknown function (φPSA17 cf. φPSA2 (38% identity/54% similarity) and φPSA17 cf. gh-1 (52% identity/69% similarity) at amino acid level). Finally, VO98_215 (T7 genes *17*) and VO98_220 (hypothetical) from φPsa17 are conserved with φPSA2, but gh-1 is less conserved, particularly at the C-terminus of VO98_215. This is consistent with the annotation of VO98_215 as the phage tail fibre protein, since gh-1 has a different host (*P. putida*) compared with the *P. syringae* pv. *actinidiae* phages φPsa17 and φPSA2. The function of VO98_220 is unknown, but interestingly, it is also conserved in *P. syringae* pv. *actinidiae* myophage φPsa374 (locus tag CF96_gp141) [27]. In summary, φPsa17 is a member of the *Autographivirinae* subfamily and *T7likevirus* genus in terms of genomic content and organisation.

### 3.4. Structural Proteome of φPsa17

**Figure 4:**
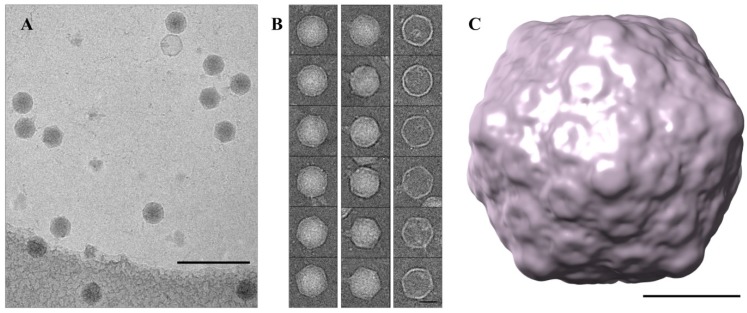
Single particle reconstructions of φPsa17. (**a**) Representative cryo-EM field of view. Scale bar 200 nm; (**b**) A gallery of cryo-EM images representing full capsids (left), capsids with visible tails (middle), and empty capsids (right), scale bar 20 nm; (**c**) 3D reconstruction of the φPsa17 phage capsid, scale bar 20 nm.

To understand how the genomic organisation relates to the phage structure, we analysed the structural proteins of purified φPsa17 phage particles using a gel-based shotgun proteomics approach. Eleven proteins were identified as structural proteins present in the φPsa17 preparations (Table 2). Each of these protein sequences was covered by more than 50% and at least 8 unique peptides. Mapping these to the translated CDSs highlighted the genes encoding the structural proteins and also indicated the abundance of the peptides detected (Figure 2A,B; purple genes and gray peptide coverage). *E. coli* phage T7 has 10 different proteins expressed as part of the class III genes and present in the mature phage structure [53]. Homologues of all of the T7 structural proteins were identified in the φPsa17 genome (Figure 2A & B) and by the proteome analysis (Table 1 & Table 2). The major capsid protein VO98_180 (T7 gp10), the internal core proteins VO98_200, VO98_205 and VO98_210 (T7 gp14, gp15 and gp16 respectively), the head-tail connector VO98_170 (T7 gp8), the non-essential head-tail protein VO98_155 (T7 gp6.7), the tail proteins VO98_185, VO98_190 and VO98_165 (T7 gp12, gp12 and gp7.3 respectively) and the tail fibre protein VO98_215 (T7 gp17) were all detected. VO98_180 appears to be the most abundant protein, consistent with its assignment as the major capsid protein, equivalent to gp10A in T7, which is present in ~415 copies [60]. No frame-shifted major capsid protein was detected (*i.e.*, the equivalent of T7 gp10B). The only other protein detected in φPsa17 was VO98_175 (T7 gp 9), albeit at the lowest sequence coverage (Table 2). Gp9 is a scaffolding protein present in T7 procapsids, but absent in mature virions [61]. Detection of VO98_175 suggested that our samples included some procapsids, some of which were detected by cryo-EM (Figure 4A & B). *E. coli* T7 and *P. putida* gh-1 both use lipopolysaccharide for attachment via the tail fibres, but the receptor for φPsa17 VO98_215 is unknown. In T7, tail (gp11 and gp12) attachment results in ejection of gp7.3 and gp6.7 into the outer membrane [53]. Upon irreversible binding, gp14, gp15 and gp16 form a channel across the cell envelope and are involved in the genome ratcheting mechanism to bring the first ~850 bp of T7 into the bacterium [53,62]. Taken together, the proteome analysis demonstrates that φPsa17 particles consist of the same structural protein complement as phage T7 and host attachment and infection are most likely analogous to T7.

**Table 2:** Identified structural proteome of φPsa17 phage particles.

Locus Tag	Description/T7 Homologue	Unique Peptides ^b^	Total Peptides	Total Mascot	Total Amanda	Total Sequest	Total PSMs ^c^	Coverage (%)
VO98_155	virion protein, gp6.7	16	18	16	14	18	426	80.00
VO98_165	tail assembly protein, gp7.3	8	10	10	6	10	140	60.00
VO98_170	collar/T7-like head-to-tail joining protein, gp8	72	112	101	70	110	2998	85.08
VO98_175	capsid and scaffold, gp9^a^	20	27	24	16	25	468	52.92
VO98_180	major capsid protein, gp10	151	215	192	69	195	7780	99.14
VO98_185	tail protein/T7-like tail tubular protein A, gp11	14	18	16	12	15	560	56.92
VO98_190	tail protein/T7-like tail tubular protein B, gp12	42	56	53	45	54	1366	71.66
VO98_200	protein inside capsid B, gp14	30	64	56	34	61	866	84.10
VO98_205	protein inside capsid C, gp15	141	221	196	131	201	4467	95.66
VO98_210	protein inside capsid D, gp16	153	232	209	165	222	4282	86.28
VO98_215	tail fibres, gp17	79	108	93	66	103	3155	92.28

^a^ In *E. coli* phage T7, gp9 is a scaffolding protein not present in the mature phage particle; ^b^ The coverage of each protein is given by the number of significantly identified unique peptides, the total number of peptides assigned and by each of the search engines (Mascot, MS Amenda, HT Sequest); ^c^ The number of acquired peptide spectra per protein (PSMs - peptide spectral matches) resulting in the identified sequence coverage (coverage (%)).

### 3.5. Cryo-TEM Structure of φPsa17

The genome and proteome analyses indicated that φPsa17 is a *T7likevirus* with similar proteins to T7 contributing to the mature virion (Figure 2). Indeed, the initial negative stained TEM results (Figure 1) indicated that φPsa17 was a member of the *Podoviridae* with a similar morphology and size to T7. A great deal of cryo-EM studies were previously published that describe the structure of the T7 phage capsid [61,63,64], the process of capsid maturation [61,65], and the conformational changes associated with genome delivery [62]. To confirm the structural morphology of φPsa17 at a higher resolution, we visualised the purified virus in a frozen hydrated state. From a total of over 200 micrographs we selected ~1400 individual particle images (e.g., Figure 4A,B). The images show capsids about 60 nm in diameter, some of them showing a clearly visible tail (Figure 4B). A small number of empty capsids were also present in the sample (Figure 4B). Based on icosahedral symmetry, we calculated a three dimensional map of the capsid to a resolution of 2.2 nm, based on the 0.143 Fourier shell correlation criterion [66]. The map presents a number of 12 prominent pentons and 20 hexons arranged in a T = 7l capsid of ~25 nm thickness and a maximum diameter of 60 nm. As expected based on the genome similarity, our reconstruction shows features similar to the T7 phage mature capsid as reported in previous studies.

## 4. Conclusions

In this study we have further characterised a podovirus that infects the economically important kiwifruit pathogen, *P. syringae* pv. *actinidiae*. A combination of next-generation genome sequencing, shotgun proteomics of structural proteins and morphological analysis by cryo-EM and single particle reconstruction revealed that φPsa17 is a member of the *T7likevirus* genus that is related to the previously sequenced pseudomonad phages φPSA2 and gh-1. T7 phages are typically thought to be obligately lytic [53], which is desirable for the development of a phage biocontrol agent. Furthermore, analysis of the φPsa17 genome did not reveal any genes involved in a lysogenic lifecycle or bacterial virulence genes. Our previous work indicated that φPsa17 has a relatively broad host-range, infecting *P. syringae* pv. *actinidiae* strains from New Zealand, Italy, Japan and South Korea and some less virulent (LV) strains from New Zealand [27], which are distinct from the virulent *P. syringae* pv. *actinidiae* isolates [20,24]. In addition, φPsa17 can infect, albeit with a lower efficiency, a non-pathogenic *P. fluorescens* isolate (ABAC62) that might be able to function as a carrier (or phage amplifying) strain [27]. Together, these features of φPsa17 indicate that it might have future potential as a biocontrol agent.
